# Alpha oscillatory dysregulation: mapping EEG oscillatory in suicidal depression

**DOI:** 10.3389/fnhum.2025.1582330

**Published:** 2025-08-11

**Authors:** Yue Zhao, Yuanyuan Guo, Dongpeng Wu, Jiahua Zhang, Shuang Zheng, Wen Xie, Kai Wang, Yanghua Tian

**Affiliations:** ^1^Department of Neurology, The First Affiliated Hospital of Anhui Medical University, Hefei, China; ^2^The College of Mental Health and Psychological Sciences, Anhui Medical University, Hefei, China; ^3^Department of Psychology, Anhui Mental Health Center, Hefei, China; ^4^Anhui Province Key Laboratory of Cognition and Neuropsychiatric Disorders, Hefei, China; ^5^Department of Neurology, The Second Affiliated Hospital of Anhui Medical University, Hefei, China

**Keywords:** depression, suicidal ideation, EEG, alpha band, power spectral density, frontal alpha asymmetry

## Abstract

**Background:**

Depression is a common mood disorder that can lead to suicide in severe cases. The aim of this study was to explore the characteristics of electrical activity in different brain regions in depressive patients with suicidal ideation (DSI), and to provide new insights into the neural mechanisms of suicidal ideation.

**Methods:**

21 DSI, 18 depressive patients without suicidal ideation (DNSI), and 20 demographically matched healthy controls (HC) were included in the study. Differences in EEG power spectral density (PSD), frontal alpha asymmetry (FAA), and functional connectivity (FC) were computationally compared among the three groups to assess the differences in these EEG metrics between the different groups.

**Results:**

EEG analysis showed a significant increase in alpha-band PSD and a significant decrease in FAA in DSI compared to DNSI (*p* < 0.05). Compared with HC, DSI exhibited a significant increase in alpha-band FC between frontal-central and parietal-central regions (*p* < 0.05). Furthermore, in DSI, alpha-band FC between frontal and central regions was significantly and positively correlated with both severity (*rho* = 0.508, *p* = 0.038) and intensity (*rho* = 0.544, *p* = 0.024) of suicidal ideation.

**Conclusion:**

This study found significant alterations in the EEG alpha band in DSI compared to DNSI and HC. alpha band alterations may be a potential biomarker of suicide risk in depression. These findings contribute to further understanding of the neural mechanisms of DSI.

## Highlights

- Alpha band power spectral density revealed the significant abnormality of depression with suicidal ideation.
- Frontal alpha band asymmetry showed a special pattern in patients with suicidal ideation.
- Functional connectivity analysis revealed abnormal alpha band connectivity in brain regions associated with suicidal ideation.
- The study provided potential biomarkers for suicide risk assessment in patients with depression.
- EEG analysis provided a new diagnostic tool and research perspective for psychiatry.

## 1 Introduction

Depression is a common mood disorder characterized by low mood and lack of motivation, which may lead to suicidal symptoms in severe cases. Several studies have shown that 18–58% of depressive patients are suicidal (Zhou et al., 2013; Sokero et al., 2003). In China, the lifetime prevalence of suicidal ideation in depressive patients is 53.1% (Dong et al., 2018). Suicidal ideation is the thought or consideration of ending one's life without taking actual action (Klonsky and May, 2014). The onset of suicidal ideation is often considered the first step in suicidal behavior and is one of the most risk factors associated with it (Klonsky and May, 2015; Dubol et al., 2018). Studies have shown that depressive patients with suicidal ideation have a higher risk of suicide compared to the general population (Zhang et al., 2020).

Currently, clinical assessment of suicidal ideation in depression relies on specific items from the Hamilton Depression Rating Scale (HAMD), the Beck Depression Inventory (BDI), the Columbia-Suicide Severity Rating Scale (C-SSRS), and the Beck Suicidal Ideation Scale (BSS). However, these scales may not accurately assess patients who are hiding suicidal ideation or exhibiting subtle symptoms. Therefore, comprehensive and accurate assessment and early intervention are key to effective suicide prevention and have important social implications. It is imperative to study the neural mechanisms of suicidal ideation in depressive patients, identify objective neurobiomarkers for early assessment, and develop specific and effective interventions.

Electroencephalography (EEG) is widely used in the study of neuropsychiatric disorders, including depression (Zhu et al., 2021), schizophrenia (Gordillo et al., 2023), epilepsy (Acharya et al., 2013), and Alzheimer's disease (Hata et al., 2016), due to its high temporal resolution, relative ease of use, low cost, and non-invasiveness (Cohen, 2017). Resting-state EEG reflects brain activity in relaxed and awake states without specific stimuli, and suicidal ideation is more likely to occur in this resting state. In fact, resting-state EEG data have shown excellent predictive ability for suicidal ideation (Benschop et al., 2019). Thus, EEG analysis is expected to provide important information related to suicide.

Power spectral density (PSD) is a commonly used EEG frequency domain analysis method that reflects the energy distribution in specific frequency bands of the brain. The alpha band has long been considered closely related to mood regulation. However, existing findings regarding alpha band power in depressive patients remain inconsistent. Some studies have found a decrease in alpha power in patients with major depression (Özçoban and Tan, 2024), while others have found an increase in alpha power in depressive patients than in healthy individuals (Jaworska et al., 2012; Grin-Yatsenko et al., 2010). Depressive patients with suicidal ideation also exhibit increased power in the occipital alpha band as well as decreased power in the beta and gamma bands (Benschop et al., 2019). These discrepancies may be attributed to differences in factors such as brain regions of interest, task or resting-state conditions, clinical characteristics (e.g., presence of suicidal ideation, depression severity), and methodological variations across studies. In addition, frontal alpha band asymmetry (FAA) has been widely used in the study of mood disorders (Mennella et al., 2017; Sharpley et al., 2023). FAA is often considered an important physiologic indicator of depression, with potential for predicting depression severity and suicide risk (Sharpley et al., 2023; Roh et al., 2020).

Meanwhile, functional connectivity (FC) is used to assess synergistic activity between different regions of the brain. Studies have shown that the pattern of functional connectivity in the brain of depressive patients is often different from that of healthy individuals (Chen et al., 2024), especially between brain regions involved in emotional and cognitive processing (Zeng et al., 2024), which may reflect abnormal information transfer processes between different brain regions. Therefore, exploring these EEG features can not only elucidate the neural mechanisms of suicidal ideation in depression, but also help to identify objective biomarkers for diagnosis or assessment, as well as provide a new research basis for early intervention and individualized treatment.

## 2 Material and methods

### 2.1 Subjects

This study included 39 patients with depression from the outpatient and inpatient departments of Anhui Mental Health Center. All patients were co-diagnosed by at least two senior psychiatrists. Inclusion criteria: (1) aged between 18 and 65 years old and habitually right-handed; (2) meeting the diagnostic criteria for depression in the Diagnostic and Statistical Manual of Mental Disorders, Fifth Edition (DSM-5); (3) being able to understand and complete the assessment scale. Exclusion criteria: (1) past or present other psychiatric disorders such as bipolar affective disorder, autism, schizophrenia, substance abuse, etc.; (2) past or present serious physical or neurological illnesses such as malignant tumors, epilepsy, etc.; (3) pregnant or lactating women; (4) physical therapy with Electroconvulsive Therapy (ECT), repetitive Transcranial Magnetic Stimulation (rTMS), or transcranial Electrical Stimulation (tES) within the past 6 months.

In addition, 20 right-handed healthy controls (HC) matched to the patient's age, gender, and years of education and with HAMD score ≤ 7 were included. The study followed the principles of the Declaration of Helsinki and was approved by the Research Ethics Committee of Anhui Medical University (approval number: 20160236). All participants were fully informed of the purpose and procedures of the study and voluntarily signed an informed consent form before participating in the study.

### 2.2 Clinical assessments

The demographic data collected for all participants included gender, age, and years of education. Epidemiological data for all patients with depression included the first episode/recurrence status, total duration of illness, current episode duration, presence of psychotic symptoms, and family history of mental illness. All participants underwent the following mood and cognitive assessments: HAMD: used to assess the severity of depressive symptoms (Hamilton, 1960). Patients with a score ≥ 2 on the third suicidal item were included in the suicidal ideation group (DSI; *n* = 21), and those with a score < 2 were categorized into the no suicidal ideation group (DNSI; *n* = 18). In the DSI group, although participants had suicidal ideation, none had resulted in serious consequences; all cases were promptly rescued, with no coma or severe outcomes, and T1-weighted MRI scans also showed no abnormalities. Hamilton Anxiety Rating Scale (HAMA): used to rate the severity of anxiety symptoms (Hamilton, 1959); C-SSRS: used to assess the severity of suicidal ideation and behavior (Posner et al., 2011); BSS: used to assess the severity of suicidal ideation (Beck et al., 1979); Montreal Cognitive Assessment (MoCA): used to assess cognitive functioning (Nasreddine et al., 2005).

### 2.3 Resting-state EEG data collection

Resting-state EEG data were collected using an EGI system (Electrical Geodesic Inc., OR, USA) with a 64-channel Ag/AgCl electrode cap, positioned according to the international 10–10 system. The reference electrode was placed at Cz, with a sampling rate of 1,000 Hz. During data collection, electrode impedance was kept below 30 kΩ. Participants sat relaxed, awake, and with eyes closed in a dimly lit, soundproof room for ~5 min.

### 2.4 Resting-state EEG data analysis

#### 2.4.1 EEG data preprocessing

Resting-state EEG data were preprocessed using the EEGLAB2022.1 toolbox in MATLAB 2023b (Delorme and Makeig, 2004). The preprocessing steps included: (1) removing irrelevant electrodes like electrooculography (EOG), leaving 60 electrodes; (2) performing a 1–50 Hz bandpass filter and a 48–52 Hz notch filter to remove powerline interference; (3) downsampling to 500 Hz; (4) interpolating bad channels and removing obvious artifacts (such as poor contact; Perrin et al., 1989); (5) re-referencing to the average reference; (6) using independent component analysis (ICA) to correct for artifacts from blinks, eye movements, muscle activity, and cardiac signals (Anemüller et al., 2003); (7) segmenting the data into 2-s epochs. Mean ± standard deviation of the number of epochs (DSI: 157.57 ± 39.83, DNSI: 160.17 ± 24.32, HC: 167.45 ± 23.73).

#### 2.4.2 PSD analysis

PSD was calculated for the preprocessed EEG data using the Welch method (Welch, 1967). For each 2-s segment, a 2-s Hamming window with 50% (1-s) overlap was used. The PSD results for each segment were averaged, and then averaged across different frequency bands and brain regions to obtain the mean PSD (in dB) for each brain region and frequency band. The frequency bands were defined as δ (1–4 Hz), θ (4–8 Hz), α (8–13 Hz), β (13–30 Hz), and γ (30–50 Hz). The 60 channels were divided into frontal, central, temporal, occipital, and parietal regions (Deng et al., 2024; Supplementary material 1).

#### 2.4.3 FAA analysis

Based on previous studies (Reznik and Allen, 2018; Gold et al., 2013; Roh et al., 2020), common channel pairs Fp1 and Fp2, F3 and F4, and F7 and F8 were selected for FAA calculation. The PSD for each channel was calculated using the Welch method, and the logarithmic difference between the left and right channel PSDs was computed as shown in Formula 1:


(1) $$\begin{array}{l} \text{FAA}=\text{log}\left(\text{Pleft}\right)\text{–log}\left(\text{Pright}\right) \end{array}$$

where Pleft and Pright represent the PSD of the left and right brain channels, respectively.

#### 2.4.4 FC analysis

FC analysis of resting-state EEG was performed using the EEGLAB2022.1 toolbox (Delorme and Makeig, 2004) and the CSD 1.1 toolbox (Kayser and Tenke, 2006). First, the current source density (CSD) for all electrodes was calculated from the preprocessed EEG data. Then, the weighted phase lag index (wPLI) was computed to measure FC (Vinck et al., 2011). The wPLI quantifies the phase difference between two time series, weighted to reduce the influence of noise, and is not affected by volume conduction effects. Finally, the average wPLI across relevant frequency bands and brain regions was calculated to obtain the FC for different brain regions and frequency bands, using the same frequency bands and brain region divisions as in the PSD analysis.

### 2.5 Statistical analysis

Statistical analyses were conducted using R Studio 4.1.2 (R Studio Inc., Boston, MA). For categorical variables such as gender and first episode/recurrence status, the Chi-square test was employed. For continuous variables such as the total duration of illness, independent samples *t*-tests (for normally distributed data) or Mann-Whitney tests (for non-normally distributed data) were used to compare differences between the DSI and DNSI groups. One-way ANOVA (for normally distributed data) or Kruskal-Wallis tests (for non-normally distributed data) were utilized to compare age, years of education, scale scores, and EEG data among the DSI, DNSI, and HC groups. If the results of one-way ANOVA or Kruskal-Wallis tests indicated significant differences, *post-hoc* analyses were performed using LSD and Dunn's tests for pairwise comparisons, with Bonferroni correction applied.

In EEG analyses, gender and age were included as covariates, and the False Discovery Rate (FDR) was employed to correct for multiple comparisons between EEG metrics (PSD and FC). A significance level of two-tailed *p* < 0.05 was set for all tests. Additionally, within the DSI group, the relationship between suicide ideation scores (C-SSRS and BSS) and the EEG metrics was examined using Spearman's correlation analysis, with gender and age as covariates, and a significance level of two-tailed *p* < 0.05.

## 3 Results

### 3.1 Demographic and clinical assessment of subjects

The study population consisted of 21 DSI, 18 DNSI, and 20 HC. The demographic and clinical information of all subjects is shown in Table 1. There were no significant differences between the three groups in terms of gender, age and years of education (*p* > 0.05). In addition, there were no significant differences between the DSI and DNSI groups in terms of first—episode/relapse status, total duration of illness, current duration of illness, presence of psychotic symptoms or family history of psychosis (*p* > 0.05).

**Table 1:** Demographic and clinical information of depressive patients with or without suicidal ideation, and healthy controls.

	**DSI**	**DNSI**	**HC**	**Statistics**	***P***
Sample size	21	18	20	NA	NA
Gender (male/female)	5/16	8/12	10/10	χ^2^ = 3.28^a, d^	0.194
Age (years)	31 (24, 51)	33.5 (28.3, 43.2)	26 (22.7, 50.5)	*H* = 0.38^b, d^	0.686
Years of education (years)	12 (8, 16)	11.5 (8, 14)	16 (11.8, 17)	*H* = 2.40^b, d^	0.100
Episode (first episode/recurrent)	12/9	8/10	NA	χ^2^ = 0.22^a, e^	0.639
Total duration of illness (months)	36 (24, 84)	36 (27, 105)	NA	*Z* = −0.47^c, e^	0.639
Current episode duration (months)	5 (2, 12)	2.5 (2, 4.75)	NA	*Z* = 1.69^c, e^	0.091
Psychiatric symptoms (present/absent)	0/21	2/16	NA	χ^2^ = 0.71^a, e^	0.401
Family history (present/absent)	2/19	1/17	NA	χ^2^ = 0.00^a, e^	1.000
**Medication**					
SSRIs	14	11	NA	NA	NA
SNRIs	8	5	NA	NA	NA
SARIs	0	0	NA	NA	NA
NaSSAs	2	4	NA	NA	NA
TCAs	4	2	NA	NA	NA
TeCAs	0	0	NA	NA	NA
APs	10	5	NA	NA	NA
ACs	9	5	NA	NA	NA
NBH	9	6	NA	NA	NA
Novel antidepressants	1	0	NA	NA	NA

^a^Chi-square test; ^b^Kruskal-Wallis test; ^c^Mann-Whitney test; ^d^Comparison among three groups; ^e^Comparison between two groups. DSI, depression with suicidal ideation; DNSI, depression without suicidal ideation; HC, healthy control; NA, not applicable/no data; SSRIs, selective serotonin reuptake inhibitors; SNRIs, serotonin-norepinephrine reuptake inhibitors; SARIs, serotonin antagonists and reuptake inhibitors; NaSSAs, noradrenergic and specific serotonergic antidepressants; TCAs, tricyclic antidepressants; TeCAs, tetracyclic antidepressants; APs, antipsychotics; ACs, anticonvulsants (mainly including valproates and benzodiazepines, used for mood stabilization or treating insomnia); NBHs, non-benzodiazepine hypnotics; novel antidepressants, including agomelatine, vortioxetine, and others.

Significant differences in HAMD, HAMA, and MoCA scores were found between the three groups (*p* < 0.05). *Post-hoc* test showed that there was a significant difference between the DSI and DNSI group on HAMD scores (*p* < 0.05), but no statistical significance after removing HAMD suicidal item score. On all these indices, there was a significant difference between the DSI group and the HC group (*p* < 0.05). There was a significant difference between the DNSI group and the HC group in HAMD, HAMA (*p* < 0.05; Table 2).

**Table 2:** Clinical assessment data of depressive patients with or without suicidal ideation, and healthy controls.

	**DSI**	**DNSI**	**HC**	**Statistics**	***P***	** $\eta_{\text{p}}^{2}$ **	***Post-hoc*** **test**			
							**Comparison**	**Statistic value**	***P***	**Cohen's** ***d***
HAMD	20 (18, 26)	13.5 (10.3, 16.5)	0 (0, 1.25)	*H* = 45.33^a^	< 0.001^***^	0.45	DSI-DNSI	*Z* = 2.45^b^	0.043^*^	1.26
							DSI-HC	*Z* = 6.68^b^	< 0.001^***^	6.11
							DNSI-HC	*Z* = 4.00^b^	< 0.001^***^	3.21
HAMD-wSI	17 (16, 22)	13.5 (10.25, 16.5)	0 (0, 1.25)	*H* = 42.62^a^	< 0.001^***^	0.43	DSI-DNSI	*Z* = 1.81^b^	0.210	0.76
							DSI-HC	*Z* = 6.38^b^	< 0.001^***^	5.62
							DNSI-HC	*Z* = 4.34^b^	< 0.001^***^	3.21
HAMA	19 (18, 23)	12.5 (8.25, 19.5)	1 (0, 2)	*H* = 42.52^a^	< 0.001^***^	0.43	DSI-DNSI	*Z* =1.83^b^	0.2028	1.02
							DSI-HC	*Z* = 6.38^b^	< 0.001^***^	5.09
							DNSI-HC	*Z* = 4.33^b^	< 0.001^***^	2.59
C-SSRS-suicidal ideation intensity	11 (11, 15)	0 (0, 0)	0 (0, 0)	NA	NA	NA	NA	NA	NA	NA
BSS-suicidal ideation	4.00 (2, 6)	0 (0, 0)	0 (0, 0)	NA	NA	NA	NA	NA	NA	NA
BSS-suicide risk	6 (3, 10)	0 (0, 0)	0 (0, 0)	NA	NA	NA	NA	NA	NA	NA
MoCA	25 (24, 26)	26 (24, 29)	27 (26, 28)	*H* = 9.04^a^	0.011	0.14	DSI-HC	*Z* = 3.01^b^	0.008^**^	1.02

^a^Kruskal-Wallis test; ^b^Post-hoc test—Dunn's test; ^*^p < 0.05; ^**^p < 0.01; ^***^p < 0.001. DSI, depression with suicidal ideation; DNSI, depression without suicidal ideation; HC, healthy control; HAMD, Hamilton depression rating scale; HAMA, Hamilton anxiety rating scale; HAMD-wSI, Hamilton depression rating scale—without suicide item; C-SSRS, Columbia-suicide severity rating scale; BSS, Beck scale for suicide ideation; MoCA, Montreal cognitive assessment; NA, not applicable/no data.

### 3.2 EEG PSD analysis results

**Figure 1:**
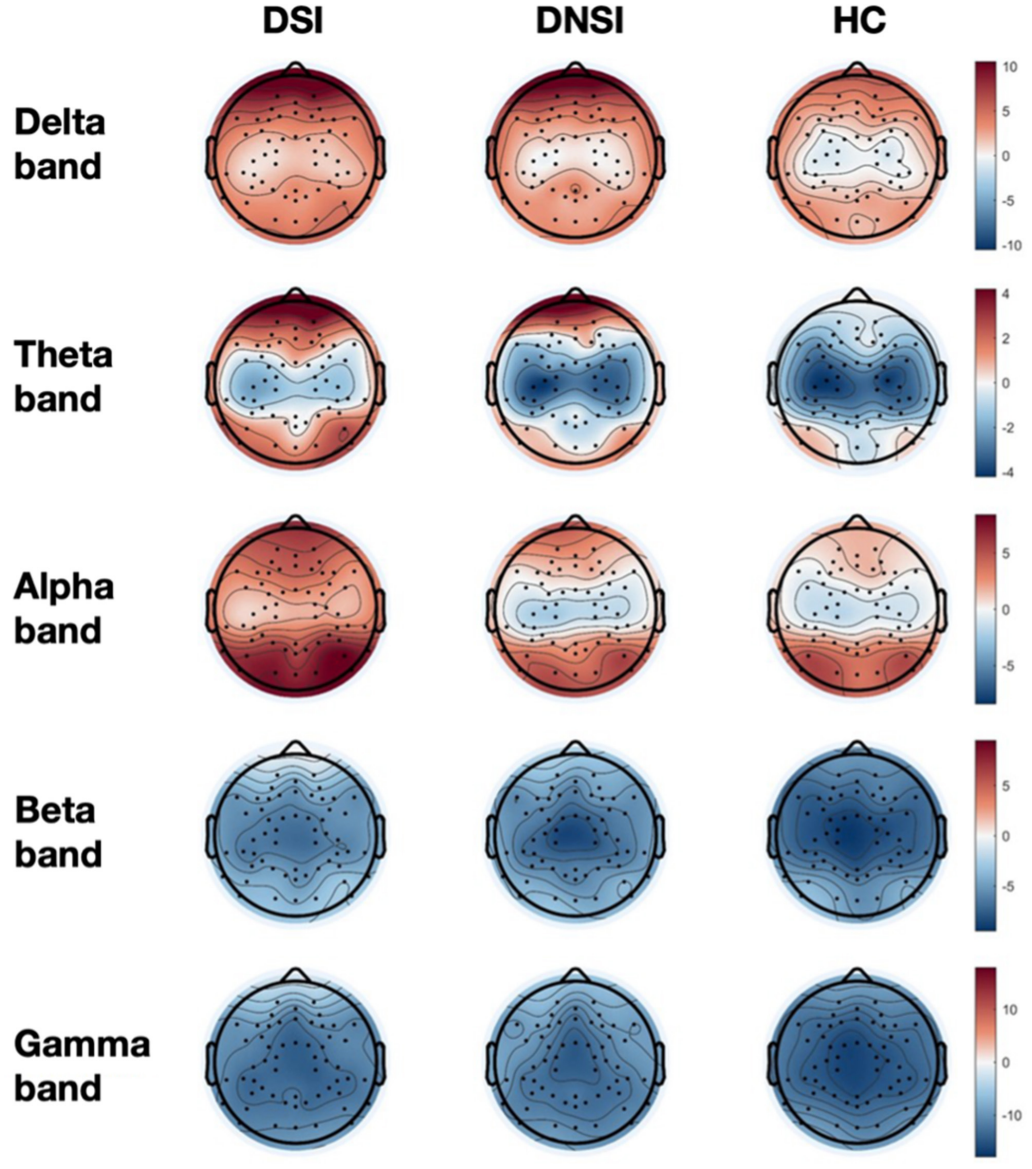
Topographical maps of EEG power spectrum density for three groups. DSI, patients with depression and suicidal ideation; DNSI, patients with depression without suicidal ideation; HC, healthy control.

This study found significant differences in the PSD among the DSI, DNSI, and HC groups in the frontal lobe across delta, theta, alpha, beta, and gamma frequency bands; in the central region across delta, theta, and alpha frequency bands; and in the temporal, parietal, and occipital lobes exclusively in the alpha frequency band (*p* < 0.05, FDR corrected). *Post-hoc* analysis revealed that the PSD values in the DSI group were higher than those in the HC group for the bands. Specifically, the DSI group exhibited significantly higher alpha band PSD across all brain regions compared to the DNSI group. Additionally, the DNSI group showed higher delta and gamma band PSD in the frontal lobe compared to the HC group (*p* < 0.05, Bonferroni corrected; Figure 1, Supplementary material 2).

### 3.3 EEG FAA analysis results

**Figure 2:**
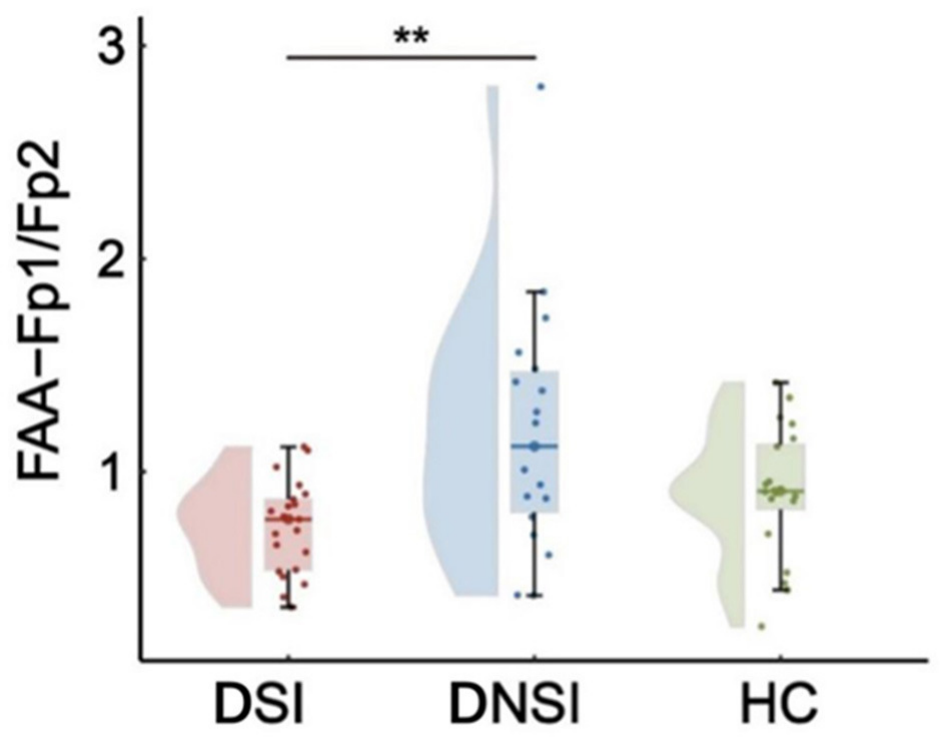
Results of EEG frontal alpha asymmetry. **p < 0.01. FAA, frontal alpha asymmetry; DSI, depression with suicidal ideation; DNSI, depression without suicidal ideation; HC, healthy control.

This study found significant differences in frontal asymmetry in the alpha frequency band (FAA) among the DSI, DNSI, and HC groups for FAA-Fp1/Fp2 (*F* = 5.76, *p* = 0.005, *p*FDR = 0.016, $\eta_{\text{p}}^{2}$ = 0.17), while no significant differences were observed for FAA-F3/F4 and FAA-F7/F8 among the three groups (*p* > 0.05, FDR corrected). *Post-hoc* analysis revealed that FAA-Fp1/Fp2 in the DSI group was significantly lower than that in the DNSI group (*t* = −3.36, *p* = 0.004, Cohen's *d* = 1.04), with no significant differences observed between DSI and HC groups or between DNSI and HC groups (Figure 2).

### 3.4 EEG FC analysis results

**Figure 3:**
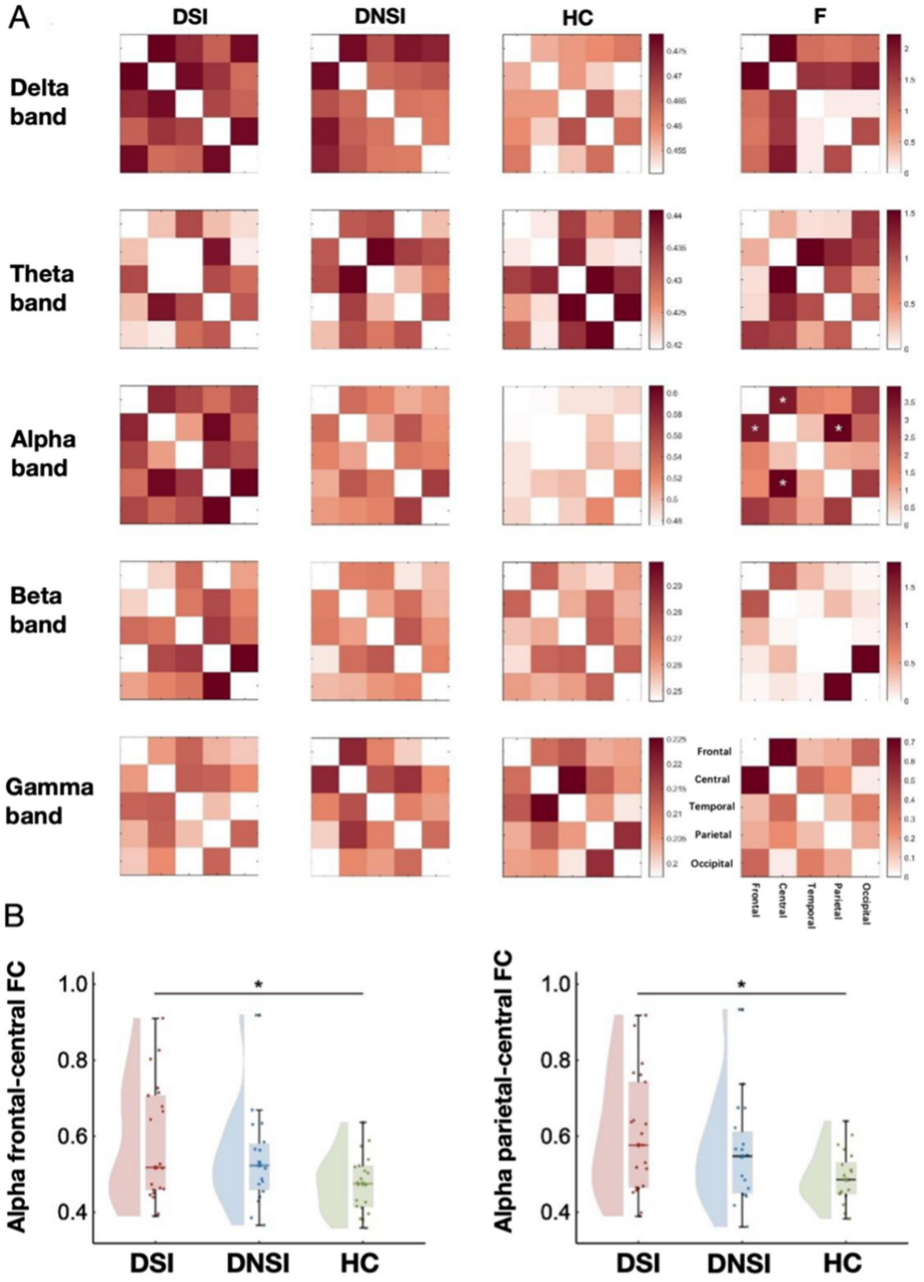
Results of EEG functional connectivity analysis. **(A)** Functional connectivity maps of each brain region in each band among the three groups and F-value maps for comparison among the three groups; **(B)** Left: statistical maps of frontal-central functional connectivity in the alpha frequency band among the three groups; right: statistical maps of central-parietal functional connectivity in the alpha band among the three groups. *p < 0.05. DSI, patients with depression with suicidal ideation; DNSI, patients with depression without suicidal ideation; HC, healthy control.

**Figure 4:**
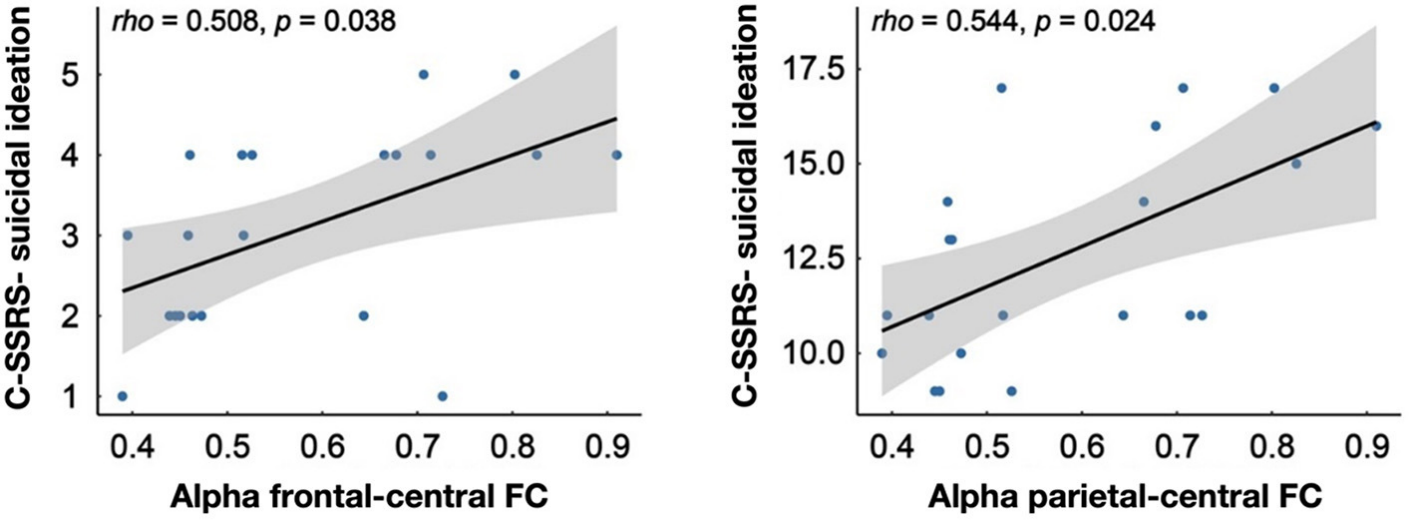
Results of correlation analysis.

This study found significant differences in FC in the alpha frequency band between frontal-central regions (*F* = 3.44, *p* = 0.039, *p*FDR = 0.838, $\eta_{\text{p}}^{2}$ = 0.11) and parietal-central regions (*F* = 3.95, *p* = 0.025, *p*FDR = 0.838, $\eta_{\text{p}}^{2}$ = 0.13) among the DSI, DNSI, and HC groups (Figures 3, 4). *Post-hoc* analysis revealed that only the DSI group showed significantly higher FC compared to the HC group in these connections (frontal-central: *t* = 2.62, *p* = 0.034, Cohen's *d* = 0.86; parietal-central: *t* = 2.81, *p* = 0.021, Cohen's *d* = 0.88). There were no significant differences between DSI and DNSI groups or between DNSI and HC groups (Figure 3).

### 3.5 Correlation analysis results

The correlation analysis revealed significant positive correlations within the DSI group between the alpha-band FC of the frontal lobe to the central region and the degree of suicidal ideation measured by C-SSRS (*rho* = 0.508, *p* = 0.038) as well as the intensity of suicidal ideation measured by C-SSRS (*rho* = 0.544, *p* = 0.024; Figure 4).

## 4 Discussion

This study examined the EEG characteristics in depressive patients with suicidal ideation, including power spectral density, frontal alpha asymmetry, and functional connectivity and incorporating neuropsychological assessments. The findings revealed significant abnormalities in alpha activity across specific brain regions in depressive patients with suicidal ideation.

This study found significant differences in HAMD scores between DSI and DNSI, and there was no significant difference in HAMD-without Suicide Item (HAMD-wSI), indicating that the two groups of patients may have similar degrees of other depressive symptoms except suicidal ideation. This suggests that suicidal ideation may play an important role in the evaluation of clinical severity of depression, and the study of depression with suicidal ideation plays an important role in the risk stratification of depression. In addition to emotional symptoms, this study also found that the overall cognitive function of SI patients was worse than that of HC, while there was no significant difference between NSI patients and HC, which was consistent with previous studies on the high correlation between suicidal ideation and cognitive impairment (Barzilay et al., 2019; Pu et al., 2017), and longitudinal studies also found that the improvement of verbal memory was related to the decline of suicide scores. Independent of improvement in depressive symptoms (Pu et al., 2017), this suggests a strong link between cognitive function and suicidal ideation, possibly due to increased neurological atrophy caused by reduced expression of Brain-Derived Neurotrophic Factor (BDNF) in SI patients. And inhibits neurogenesis in the prefrontal cortex-limbic brain region, which regulates parts of cognitive function such as executive function and memory (Gorlyn et al., 2015; Duman and Monteggia, 2006).

The study found a significant increase in alpha-band PSD in DSI compared to DNSI based on EEG analysis. Similar results have been found in previous studies, and a cross-sectional EEG study found a significant increase in alpha-band power in the occipital region in patients with DSI compared to DNSI (Benschop et al., 2019). In addition, a sleep EEG study found increased alpha-band power in depressed patients with high suicidal ideation compared to those with low suicidal ideation (Dolsen et al., 2017). There was also an intervention study that found a decrease in suicidal ideation and alpha band power in DSI after ketamine treatment. The alpha band is involved in emotion regulation. Impairment of the occipital alpha network has been found to be associated with impaired processing of emotional stimuli (Chao et al., 2022; Godsil et al., 2013). Abnormal coordination between delta activity in the frontal lobe and alpha activity in the occipital region may lead to pleasure deficit and rumination symptoms (Godsil et al., 2013), both of which are closely related to suicidal ideation.

The study further found that FAA was significantly lower in DSI than in DNSI. The lower FAA indicated a relative enhancement of alpha-band power in the left frontal region, suggesting reduced cortical activity in the left frontal lobe (Bruder et al., 2017). Reduced cortical activity in the left ventral lateral prefrontal lobe has been associated with decreased pleasure, which can further contribute to symptoms of pleasure deficit (Gollan et al., 2014; Meerwijk et al., 2013). Abnormal activity in the left dorsolateral prefrontal cortex may inhibit positive stimuli while hindering inhibition of negative stimuli, thus promoting a tendency to ruminate (Trew, 2011; Meerwijk et al., 2013). Defective functioning of the left orbitofrontal cortex has been associated with impulsivity (Disner et al., 2011; Best et al., 2002), all of which further increases suicidal ideation and suicide risk (Treadway and Zald, 2011; Smith et al., 2006; Dougherty et al., 2004). Similar results have been found in previous studies; it was found that suicidal depressed adolescents had significantly higher power in the left alpha band than in the right (Graae et al., 1996). In summary, these findings suggest that the alpha band plays a crucial role in the neural mechanisms of depression with suicidal ideation.

In addition, this study found a significant increase in functional connectivity within the alpha band between the frontal-central and parietal-central regions in DSI patients. The frontal lobe plays a crucial role in emotion regulation and higher-order cognitive functions (Catani, 2019). The central region is involved in the integration of sensory and motor functions (Raza et al., 2015; Yuan et al., 2023). The enhanced functional connectivity suggests tighter information transfer between the frontal and central regions, potentially indicating that DSI patients require more resources to manage emotions. The parietal lobe is responsible for spatial perception and attention regulation (Bisley and Goldberg, 2010), and depressed patients are often more sensitive to negative information in their environment (Reinen et al., 2021). In patients with depression and suicidal ideation, increased parietal-central connectivity may reflect heightened sensitivity to external stimuli.

Moreover, further analyses showed that alpha-band FC between the frontal lobe and the central region was significantly correlated with the degree and intensity of suicidal ideation, suggesting that the neural activity in this region may have an important role in the assessment of suicidal risk. Such connectivity changes may reflect the complex neural mechanisms involved in the generation and maintenance of suicidal ideation, suggesting that we should consider these neurobiological indicators in the development of intervention strategies.

## 5 Limitations

This study also has several limitations. First, the sample size of this study is relatively small. Future studies will aim to expand the sample size to enhance the robustness of the findings. Second, this study only analyzed EEG data in the closed-eye resting state. Future studies may incorporate EEG in the eyes-open resting state to fully explore the neural mechanisms of depression with suicidal ideation. Finally, considering that patients with suicidal ideation usually require medication, future studies will include those who have been on a stable medication regimen for a certain period of time.

## 6 Conclusion

In summary, this study found through resting-state EEG frequency spectrum analysis that DSI exhibited significantly increased alpha band PSD and FC abnormalities, along with significantly decreased FAA. These results indicate the crucial role of the alpha band in the symptoms of suicidal ideation in depression, providing valuable insights for future non-invasive physical interventions aimed at regulating suicidal ideation symptoms.

## Data Availability

The raw data supporting the conclusions of this article will be made available by the authors, without undue reservation.

## Supplementary material

Supplementary material 1 EEG channel brain region division diagram.

Supplementary material 2 Results of EEG power spectrum density analysis.
