# Supplementary figures and images for: Alpha oscillatory dysregulation: mapping EEG oscillatory in suicidal depression
Source: Front Hum Neurosci. 2025 Aug 11;19:1582330. doi: 10.3389/fnhum.2025.1582330 (PMC12375628; doi:10.3389/fnhum.2025.1582330)

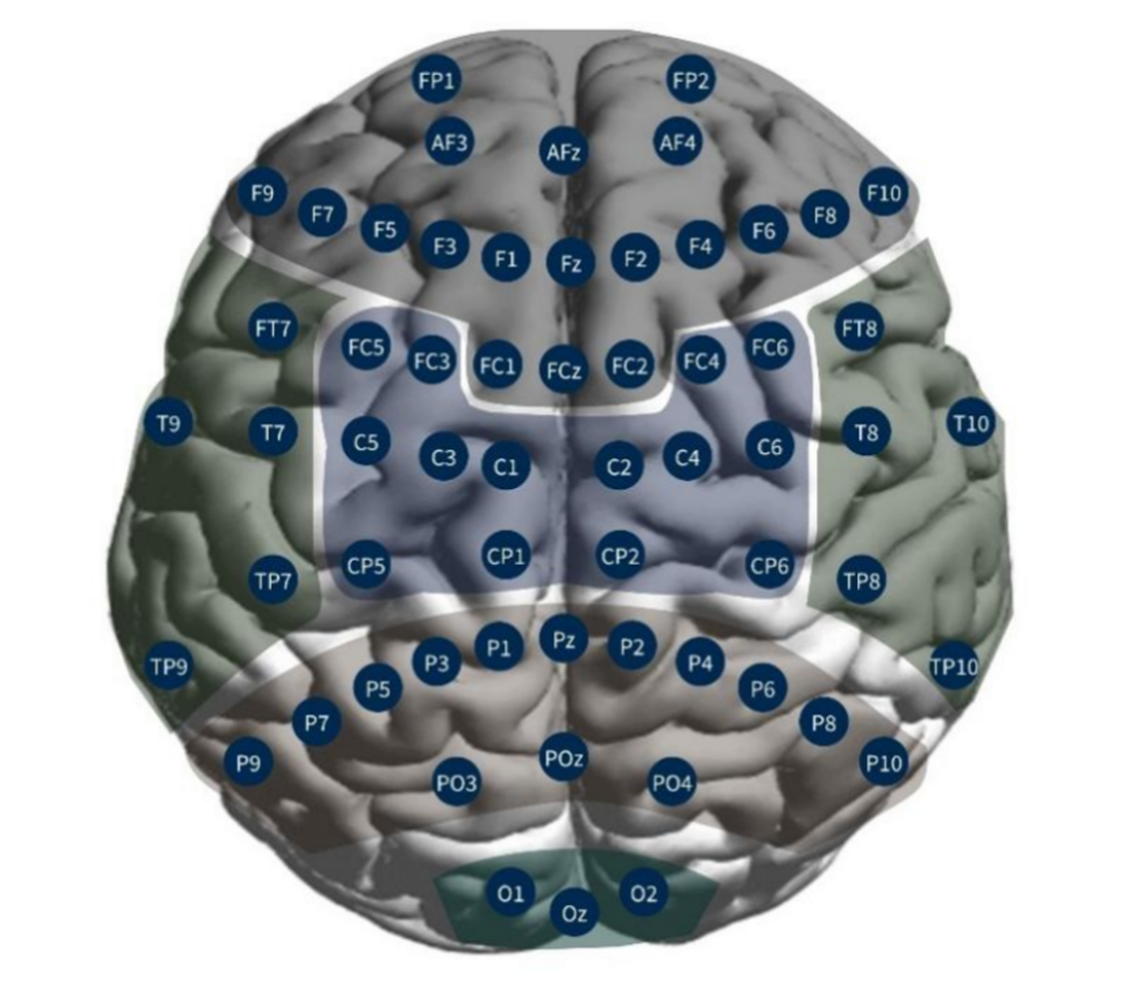

Supplement: Supplementary material 1 — EEG channel brain region division diagram. [file Image_1.tiff]
