# Supplementary material for: Alpha oscillatory dysregulation: mapping EEG oscillatory in suicidal depression
Source: Front Hum Neurosci. 2025 Aug 11;19:1582330. doi: 10.3389/fnhum.2025.1582330 (PMC12375628; doi:10.3389/fnhum.2025.1582330)
Supplement: Supplementary material 2 — Results of EEG power spectrum density analysis. [file Table_1.docx]

Supplementary material 2. Results of EEG Power Spectrum Density Analysis

|  | DSI | DNSI | HC | Statistics | *P* | P (FDR corrected) | *η*_p_^2^ | Post-hoc Test | | | |
| --- | --- | --- | --- | --- | --- | --- | --- | --- | --- | --- | --- |
|  |  |  |  |  |  |  |  | Comparison | Statistic Value | *P* | *Cohen’s d* |
| Delta Frequency |  |  |  |  |  |  |  |  |  |  |  |
| Frontal Lobe | 5.40 ± 2.60 | 4.59 ± 3.12 | 2.62 ± 1.75 | *F* = 6.74 | 0.002** | 0.015* | 0.20 | DSI-HC | *t* = 3.58 | 0.002** | 1.25 |
|  |  |  |  |  |  |  |  | DNSI-HC | *t* = 2.48 | 0.049* | 0.79 |
| Central Region | 1.41 ± 2.30 | 0.60 ± 3.26 | -0.49 ± 1.48 | *F* = 4.58 | 0.015* | 0.033* | 0.14 | DSI-HC | *t* = 3.02 | 0.011* | 0.98 |
| Temporal Lobe | 3.36 ± 2.28 | 3.17 ± 3.56 | 1.91 ± 1.69 | *F* = 2.50 | 0.092 | 0.135 | 0.08 | NA | NA | NA | NA |
| Parietal Lobe | 2.96 ± 2.88 | 2.64 ± 3.54 | 1.86 ± 1.82 | *F* = 1.13 | 0.331 | 0.360 | 0.04 | NA | NA | NA | NA |
| Occipital Lobe | 3.77 ± 3.23 | 2.98 ± 3.32 | 2.29 ± 1.84 | *F* = 1.60 | 0.211 | 0.263 | 0.05 | NA | NA | NA | NA |
| Theta Frequency |  |  |  |  |  |  |  |  |  |  |  |
| Frontal Lobe | 1.31 ± 2.18 | -0.138 ± 2.53 | -1.29 ± 2.23 | *F* = 6.00 | 0.004** | 0.022* | 0.18 | DSI-HC | *t* = 3.46 | 0.003** | 1.18 |
| Central Region | -1.30 ± 2.54 | -3.12 ± 2.64 | -3.48 ± 2.34 | *F* = 4.80 | 0.012* | 0.031* | 0.15 | DSI-HC | *t* = 2.93 | 0.015* | 0.89 |
| Temporal Lobe | 0.41 ± 2.22 | -0.86 ± 2.90 | -1.62 ± 2.59 | *F* = 3.40 | 0.041* | 0.079 | 0.11 | NA | NA | NA | NA |
| Parietal Lobe | 0.68 ± 2.95 | -0.61 ± 2.75 | -1.10 ± 2.64 | *F* = 2.06 | 0.138 | 0.182 | 0.07 | NA | NA | NA | NA |
| Occipital Lobe | 1.46 ± 3.07 | 0.01 ± 2.81 | -0.36 ± 2.62 | *F* = 2.05 | 0.139* | 0.182 | 0.07 | NA | NA | NA | NA |
| Alpha |  |  |  |  |  |  |  |  |  |  |  |
| Frontal Lobe | 3.98 ± 2.06 | 1.21 ± 2.72 | 0.69 ± 3.38 | *F* = 7.01 | 0.002** | 0.015* | 0.20 | DSI-DNSI | *t* = 2.89 | 0.017* | 1.16 |
|  |  |  |  |  |  |  |  | DSI-HC | *t* = 3.49 | 0.003** | 1.18 |
| Central Region | 2.02 ± 2.33 | -1.27 ± 2.80 | -0.92 ± 3.42 | *F* = 6.94 | 0.002** | 0.015* | 0.20 | DSI-DNSI | *t* = 3.35 | 0.004** | 1.29 |
|  |  |  |  |  |  |  |  | DSI-HC | *t* = 3.06 | 0.010* | 1.01 |
| Temporal Lobe | 3.50 ± 1.72 | 0.81 ± 2.72 | 0.77 ± 3.44 | *F* = 5.70 | 0.006** | 0.024* | 0.17 | DSI-DNSI | *t* = 2.88 | 0.017* | 1.20 |
|  |  |  |  |  |  |  |  | DSI-HC | *t* = 2.94 | 0.014* | 1.01 |
| Parietal Lobe | 5.97 ± 2.34 | 2.84 ± 3.60 | 3.01 ± 3.75 | *F* = 4.96 | 0.011* | 0.031* | 0.15 | DSI-DNSI | *t* = 2.74 | 0.025* | 1.05 |
|  |  |  |  |  |  |  |  | DSI-HC | *t* = 2.69 | 0.028* | 0.95 |
| Occipital Lobe | 7.33 ± 2.22 | 4.31 ± 3.80 | 4.23 ± 3.59 | *F* = 5.47 | 0.007** | 0.024* | 0.16 | DSI-DNSI | *t* =2.72 | 0.027* | 0.99 |
|  |  |  |  |  |  |  |  | DSI-HC | *t* = 2.98 | 0.013* | 1.05 |
| Beta Frequency |  |  |  |  |  |  |  |  |  |  |  |
| Frontal Lobe | -4.63 ± 2.48 | -5.34 ± 1.94 | -6.97 ± 2.43 | *F* = 4.78 | 0.012* | 0.031* | 0.14 | DSI-HC | *t* = 3.05 | 0.011* | 0.95 |
| Central Region | -6.20 ± 2.99 | -7.07 ± 2.36 | -8.11 ± 2.87 | *F* = 2.49 | 0.092 | 0.135 | 0.08 | NA | NA | NA | NA |
| Temporal Lobe | -4.93 ± 2.40 | -5.20 ± 2.08 | -6.54 ± 2.56 | *F* = 2.86 | 0.066 | 0.110 | 0.09 | NA | NA | NA | NA |
| Parietal Lobe | -4.60 ± 2.47 | -5.45 ± 2.14 | -5.86 ± 2.48 | *F* = 1.21 | 0.306 | 0.348 | 0.04 | NA | NA | NA | NA |
| Occipital Lobe | -3.60 ± 2.67 | -4.27 ± 2.88 | -5.02 ± 2.28 | *F* = 1.34 | 0.270 | 0.321 | 0.05 | NA | NA | NA | NA |
| Gamma |  |  |  |  |  |  |  |  |  |  |  |
| Frontal Lobe | -10.40 ± 3.20 | -10.20 ± 2.62 | -13.10 ± 1.83 | *F* = 7.18 | 0.002** | 0.015* | 0.21 | DSI-HC | *t* = 3.27 | 0.006** | 1.03 |
|  |  |  |  |  |  |  |  | DNSI-HC | *t* = 3.27 | 0.006** | 1.28 |
| Central Region | -13.00 ± 4.40 | -12.30 ± 3.92 | -15.00 ± 3.26 | *F* = 3.20 | 0.049* | 0.087 | 0.10 | NA | NA | NA | NA |
| Temporal Lobe | -11.40 ± 3.78 | -10.30 ± 3.15 | -12.70 ± 2.75 | *F* = 3.46 | 0.039* | 0.079 | 0.10 | NA | NA | NA | NA |
| Parietal Lobe | -12.40 ± 2.95 | -12.20 ± 2.87 | -13.20 ± 2.11 | *F* = 0.98 | 0.381 | 0.397 | 0.03 | NA | NA | NA | NA |
| Occipital Lobe | -10.60 ± 4.14 | -10.80 ± 4.58 | -11.20 ± 3.32 | *F* = 0.25 | 0.782 | 0.782 | 0.01 | NA | NA | NA | NA |

Notes：* , *p* < 0.05; ** , *p* < 0.01. DSI, Depression with Suicidal Ideation; DNSI, Depression without Suicidal Ideation; HC, Healthy Control; NA, Not Applicable/No Data.
